# Phadiatop Infant in the Diagnosis of Atopy in Children with Allergy-Like Symptoms

**DOI:** 10.1155/2009/460737

**Published:** 2009-08-24

**Authors:** Ragnhild Halvorsen, Åsa Jenner, Else Marie Hagelin, Magnus P. Borres

**Affiliations:** ^1^Voksentoppen Center for Asthma, Allergy and Chronic Lung Diseases, University Hospital, 0791 Oslo, Norway; ^2^Phadia AB, 75137 Uppsala, Sweden; ^3^Department of Pediatrics, Sahlgrenska Academy of Göteborg University, 41685 Göteborg, Sweden

## Abstract

*Background and Objective*. Allergy-like symptoms such as wheezing and eczema are common in young children and an early diagnosis is important to initiate correct management. The objective of this study was to evaluate the diagnostic performance of Phadiatop Infant, an in vitro test for determination of early sensitisation to food and inhalant allergens. *Patients and Methods*. The study was conducted, retrospectively, using frozen sera from 122 children (median age 2.7 years) admitted to the hospital with suspected allergic symptoms. The doctor's diagnosis atopic/nonatopic was based on routinely used procedures such as clinical evaluation, SPT, total and allergen-specific IgE antibodies. The performance of Phadiatop Infant was evaluated in a blinded manner against this diagnosis. *Results*. Eighty-four of the 86 children classified as atopic showed a positive Phadiatop Infant test. Thirty-six were classified as nonatopic, 32 of who had a negative test. With a prevalence of atopy of 70% in this population, this gives a sensitivity of 98%, a specificity of 89%, and a positive and negative predictive value of 95% and 94%, respectively. *Conclusion*. The results from the present study suggest that Phadiatop Infant could be recommended as a complement to the clinical information in the differential diagnosis on IgE-mediated disease in young children with allergy-like symptoms.

## 1. Introduction

Allergic diseases are among the most common chronic diseases throughout the world [1] and the prevalence of atopic diseases in childhood has significantly increased during the past several decades [2, 3]. Although there is a general consensus on the importance of a genetic predisposition for atopic diseases, only changes in environmental factors can explain this increase [4–6].

There is a strong association between sensitisation and symptoms of allergic diseases although this association is not absolute. The “allergy march” refers to the natural history of sensitisation to allergens and symptoms of eczema, asthma, and rhinitis, which is characterized by a typical sequence of sensitisation and manifestations of symptoms that appear during a certain age period [7, 8].

Food allergy and atopic eczema during the first years of life have been considered risk factors for subsequent asthma and rhinitis caused by indoor and outdoor inhalant allergens [5, 9, 10].

However, allergy-like symptoms in childhood like wheezing and eczema need not have an atopic background [11, 12]. Wheezing episodes in young children are often transient and associated with viral respiratory infections [13, 14].

Thus, better predictors of disease development and diagnostic markers for allergic disease are needed to give proper treatment and valuable advice, especially concerning environmental control to parents of young children [15].

The present study was carried out in order to evaluate the diagnostic performance of Phadiatop Infant, an in vitro test designed to detect allergen-specific IgE antibodies known to be relevant in the development of IgE-sensitisation in early childhood.

## 2. Patients and Methods

### 2.1. Patients

The study was conducted retrospectively in consecutively included children, below the age of five years and admitted to BKL Voksentoppen, Rikshospitalet, Oslo, during a period of twelve months. The children were referred from paediatricians and other paediatric departments at hospitals in Norway and thus selected for more severe allergic symptoms. Frozen-serum samples from 122 children were analysed and clinical data documented in the patients' records were compiled for the study.

The study conformed to the principles of Helsinki's declaration and was approved by the local Ethics Committee.

### 2.2. Skin Prick Test

Skin Prick Test (SPT) was performed according to standard procedure at the hospital. Glycerinated allergen extract (Soluprick ALK-Abello, Denmark and a standard lancet with 1mm tip and a shoulder (ALK-Abello, Denmark) was used. Histamine chloride 10 mg/mL was used as positive-control and as negative-control glycerol. The reaction was recorded as 3+ when the reaction was equal to the histamine control, 2+ when half the histamine control, and 4+ when double the histamine control. The panel included egg white, cow's milk, cod fish, peanut, hazel nut, wheat, house dust mite, cat dander, birch, timothy, Cladosporium, and latex.

### 2.3. Total and Allergen-Specific IgE Antibodies

Total and allergen-specific IgE antibodies were determined in ImmunoCAP 100 (Phadia AB, Sweden) at the hospital laboratory when appropriate according to clinical findings. The instructions from the manufacturer were followed. The same allergen panel as for SPT was analysed.

### 2.4. ImmunoCAP Phadiatop, fx5, and ImmunoCAP Phadiatop Infant

Serum samples were sent to Phadia AB, Uppsala, Sweden, for determination of specific IgE antibodies using ImmunoCAP Phadiatop, fx5 (a food mixture of milk, egg white, fish, peanut, wheat, and soy bean), and ImmunoCAP Phadiatop Infant using ImmunoCAP100 (Phadia AB, Uppsala, Sweden). Results ≥ 0.35 *k*_*A*_/l were considered positive.

Phadiatop Infant is designed to detect allergen-specific IgE antibodies to food and inhalant allergens, relevant in the development of IgE-mediated disease in young children. The following allergens are included in the test: hen's egg, cow's milk, peanut, shrimp, cat epithelium and dander, dog dander, house dust mite, common silver birch, timothy, ragweed, and wall pellitory. The laboratory analyses were performed in a blinded manner and the results of Phadiatop and fx5 were sent back to the investigating physician.

### 2.5. Diagnostic Procedures

Atopy was defined as a constitutional disposition to produce IgE antibodies to common allergens whether the patients had clinical symptoms or not, as judged by the Investigator. The study was designed to make the diagnosis with best possible available tools (case history, SPT, and allergen-specific IgE results) to evaluate the clinical performance of Phadiatop Infant.

A preliminary diagnosis whether a child was atopic or not was assessed by the investigating physician in retrospect, taking into account clinical history and diagnostic procedures, which includes available SPT and allergen-specific IgE results, noted in the patient records. In the final diagnosis of the atopic state, the laboratory results from Phadiatop and fx5 were also taken into consideration in order to get comparable data of allergen-specific IgE sensitisation on all children. This final diagnosis was used as the reference for calculation of the diagnostic performance of Phadiatop Infant.

### 2.6. Statistical Analysis

Processing of data and statistical analyses were performed using SAS statistical software system. Demographic data were analysed descriptively. Quantitative variables are described in appropriate measures of localisation and dispersion, quantitatively variables presented by counts and percentages. The diagnostic performance of Phadiatop Infant was evaluated by calculating sensitivity, specificity, negative and positive predictive values (NPV, PPV), respectively, with 95% confidence intervals. The subgroups divided by age, <2 years and ≥2 years were compared with regard to the diagnostic performance of Phadiatop Infant using descriptive statistics.

## 3. Results

The demographic characteristics of the children classified according to the final diagnosis of atopy/nonatopy or inconclusive diagnosis are shown in Table 1. Thirty-eight (31%) subjects in the study population were girls and 84 (69%) were boys with a median age of 2.7 years. Of the 122 children, 86 (70%) were atopic, 26 girls and 60 boys, which is a commonly found gender distribution among atopic children at that age. No difference in median age was observed between the atopic and nonatopic children.

Only 18% of the children presented with wheezing as a single symptom and the majority of these children were nonatopic, 55% below 2 years and 40% above this age, respectively. Eczema as a single symptom was the most prevalent symptom among the children below two years of age (49%) and 63% were classified as atopic. In the older age group of children, eczema and/or wheezing in combination with other allergic symptoms dominated (41%) and 48% were classified as atopic. Other allergy-like symptoms such as rhinitis, rhinoconjunctivitis, anaphylaxis, and gastrointestinal symptoms were registered in 31 (26%) children, out of whom 22 (71%) were older than 2 years (data not shown).

Eighty-three of the children (68%) reported at least one first degree relative, with about the same proportion for the atopic as for the nonatopic children, 71% and 61%, respectively (data not shown).

The diagnostic performance characteristics of Phadiatop Infant in this study population with a prevalence of 70% are presented in Table 2. The sensitivity calculated for the whole group of children was 98% (95% CI: 92–100%) and the specificity 89% (95% CI: 74–97%). The PPV and NPV values were 95% (95% CI: 89–99%) and 94% (95% CI: 80–99%), respectively. The diagnostic performance of the test was found to be similar when the children were separated in the two age groups, below or above two years, but due to small numbers of children in the separated age groups, the calculated values are not presented.

## 4. Discussion

Symptoms of allergic disease in young children are generally unspecific and the diagnosis without objective tests could be an arbitrary process. The paediatric section of the European Academy of Allergy and Clinical Immunology has recently published a position paper with recommendations on allergy testing in children to improve the identification of allergy and quality of care [16].

An earlier published study has shown that 76 out of 147 children could not be classified as having an IgE-mediated disease or not, based on case history and physical examination alone. Allergen-specific IgE tests reduced this number to 8 [17]. Similar results were found in a recently published study, where measurements of IgE-antibodies, added to case history and physical examinations, highly improved the discrimination between IgE- and non-IgE-mediated diseases in young children [18]. The results from our study confirm these findings and suggest that Phadiatop Infant could be a useful tool for discrimination between atopy/non-atopy. A positive Phadiatop Infant test should however be followed by allergen-specific antibody testing to a selected panel to identify the offending allergen(s) [19, 20].

The test seems to be at least as useful among the youngest children, below two years, as among children at 2–4 years of age. The youngest child in the study was 6 months, which confirms findings from other publications that allergen-specific IgE-antibodies can be detected early in life [17, 18, 21]. These findings support the value of testing children with allergy-like symptoms at an early age.

Family history has been considered an important risk factor for atopy and has been widely used for prediction of allergy and allergic diseases [9]. The results from our study are in good agreement with the notion that allergy/atopy is extremely commonly found. On the other hand, the fact that 30% of the atopic children did not have a family history of allergy agrees with investigators claiming that a great portion of newly diagnosed allergics/atopics do not have a family history of allergy/atopy [4]. Thus it could be stated that family history is no longer practical in predicting allergic disease.

This study population represents a selected cohort as Voksentoppen is a hospital having referred patients with allergy-like symptoms and allergic diseases from general paediatricians in the whole country, often of a more severe character. This explains the unusual high prevalence of atopy, 70%, in this population. However, other studies have shown that Phadiatop Infant could be applied in populations with a lower prevalence of atopy, still demonstrating good performance characteristics [17, 18, 21].

The clinical appearance of allergy in our study is in agreement with the concept of the allergy march and supports what has been reported earlier from other studies [7, 8]. Eczema was the predominating symptom among the atopic children below 2 years. Thus, eczema was not common in the nonatopic group of children and only one of the 31 children with eczema was classified as nonatopic. The progression from eczema to other allergic problems was also demonstrated in this study. Eighty-six percent of the children with eczema and wheezing in combination with other symptoms were found in the older age group and as many as 82% were classified as atopic.

The majority of children, all ages, presenting with wheezing were nonatopic (73%). Many infants and children who wheeze have transient conditions associated with diminished airway function and do not have an increased risk of asthma later in life. However, children with persistent wheezing, starting during the first years of life, and with an atopic heredity, should be considered being at risk for asthma later during childhood [11, 22, 23]. Therefore, an early diagnosis of IgE sensitisation may be important for the choice of treatment to wheezing toddlers.

Children with allergic symptoms usually present at a general paediatrician who needs to discriminate which patients have to be sent to an allergist for further evaluation. Possible diagnostics interventions to avoid unnecessary referrals are discussed in a recently published paper. Rule-out tests with a high discriminating potential are suggested to have a gateway function to fulfil this differentiation and an important role to prevent the march of allergic children from the first to secondary level of care [24].

The present study shows that Phadiatop Infant has a diagnostic performance with a high sensitivity and specificity. Thus, the test could be recommended as a complement to the clinical information in the differential diagnosis on IgE-mediated disease in young children with allergy-like symptoms. Furthermore, the test could be useful in assisting primary care physicians in selecting atopic children at an early stage for further intervention or referral to an allergist.

An early correct diagnosis will thus allow for better management and a possibility to delay or even prevent the onset of asthma in children with eczema and the avoidance of further deterioration of lung function in children with asthma [16, 25].

## Figures and Tables

**Table 1:** Distribution of patients by atopic classification, gender and age.

Patient group	Total *n* (%)	Gender female/male *n* (%)	Age years median (min-max)
Atopic	86 (70)	26 (30)/60 (70)	2.7 (0.7–4.7)
Nonatopic	36 (30)	12 (33)/24 (67)	2.6 (0.5–4.6)

Total	122 (100)	38 (31)/84 (69)	2.7 (0.5–4.7)

**Table 2:** Diagnostic performance characteristics of Phadiatop Infant. Data are given as number of children.

Phadiatop Infant test result	Total	All ages		<2 years		≥2 years	
		Atopic**	Nonatopic**	Atopic**	Nonatopic**	Atopic**	Nonatopic**
Positive	88	84	4	30	1	54	3
Negative	34	2	32	0	9	2	23

Total	122	86	36	30	10	56	26

**Atopic/nonatopic was classified prior to Phadiatop Infant was analysed.
